# Endovascular Reparation of Central Vein Injury with Balloon-Protected Embolization

**DOI:** 10.1007/s00270-014-0945-7

**Published:** 2014-07-04

**Authors:** Jerzy Garcarek, Ewa Wątorek, Jacek Kurcz, Mariusz Kusztal, Tomasz Gołebiowski, Krzysztof Letachowicz, Waldemar Letachowicz, Wacław Weyde, Marian Klinger

**Affiliations:** 1 Department of Radiology, Wroclaw Medical University Borowska, 213 50-556 Wroclaw, Poland; 2 Department of Nephrology and Transplantation Medicine, Wroclaw Medical University Borowska, 213 50-556 Wroclaw, Poland

## Letter to Editor,

Iatrogenic central venous injury is a potentially life-threatening complication following central vein catheterization. The incidence of catheter misplacement is 3–4 % [1]. Mortality associated with superior vena cava rupture is reported to be 44 % [2]. Data on the methods of treatment of these serious events are scarce. The postulated approaches depend on the individual clinical conditions of the patient. Vein injury can be treated either with open surgery, videothoracoscopy, or with endovascular methods, which include mainly stent-graft implantation [2–5]. One report of direct intrapericardial thrombin administration after the perforation of pericardium by the insertion of tunneled haemodialysis catheter was noted [6]. We present a novel endovascular approach to iatrogenic central vein perforation by the use of metal coils and Histoacryl glue.

The subject was an 83-year-old female patient with end-stage renal disease, with significant comorbidities: generalized atherosclerosis, chronic cardiovascular insufficiency, history of myocardial infarction, required tunneled dialysis catheter placement due to dysfunction of the fistula. Because of the oedema of the right upper extremity with the fistula, the left internal jugular vein was chosen for catheterization. Dual-lumen, radiopaque, 14.5 F, 23-cm tip to cuff length tunneled hemodialysis catheter (Bard HemoStar) was inserted using Seldinger technique under the ultrasound guidance. After catheter insertion, the patient’s condition remained unchanged with stable blood pressure. The attempt to draw blood from both catheter lines appeared unsuccessful. In the next step, chest x-ray was performed, which showed unusual catheter position, suggesting that the catheter tip left intravascular space and entered mediastinum. Bilaterally, the fluid in pleural cavities was noted, as observed before the procedure. Contrast administration through the catheter confirmed left innominate vein perforation. As far as the vast comorbidities of our patient are concerned, we refrained from thoracotomy. The vessel injury repair with stent-graft insertion also was abandoned due to the high risk of thrombosis. Therefore, we decided to repair the vessel injury with balloon-protected Histoacryl embolization with the additional use of metal coils.

**Fig. 1:**
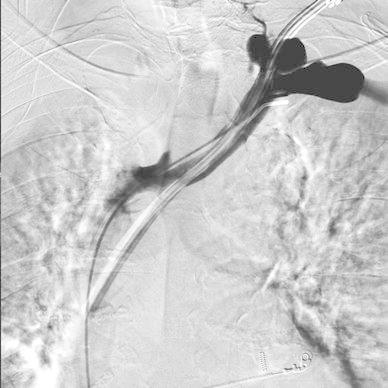
Venography of left innominate vein using catheter introduced via right common femoral vein. The site of perforation is well seen

**Fig. 2:**
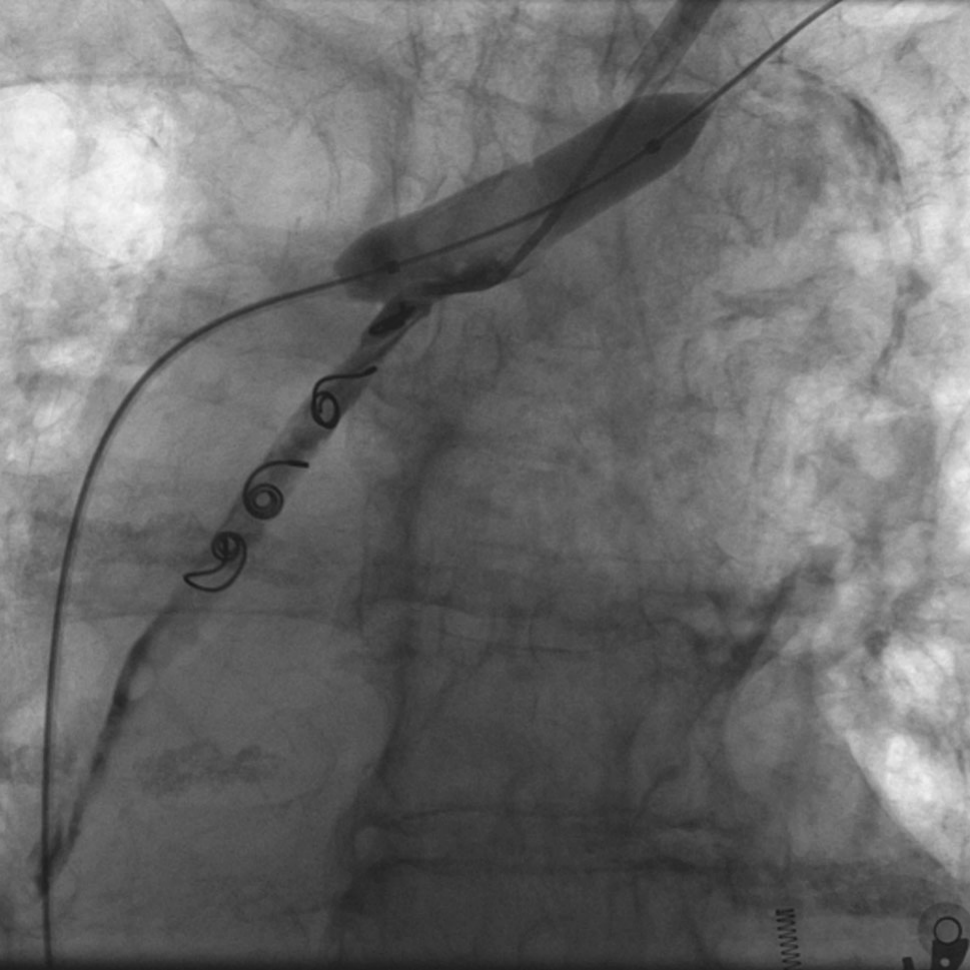
Implantation of four metal coils into the tissue canal followed by application of Histoacryl glue using catheter balloon protection

**Fig. 3:**
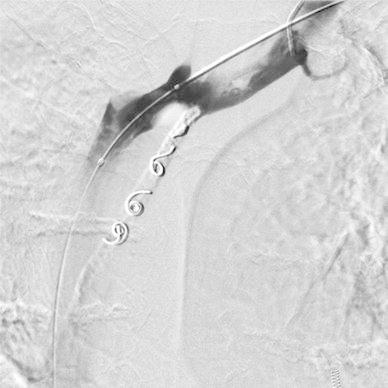
Follow-up venography. Complete occlusion of the tissue canal

Diagnostic catheter Vertebral 5F (Boston Scientific) was introduced via the right femoral vein into the left subclavian vein to carry out venography to localize the site of innominate vein perforation caused by the tunneled dialysis catheter (Fig. 1). In the next step, the diagnostic catheter was replaced by protective balloon catheter of 12 mm in diameter, 40 mm in length, Maxi LD Cordis. Then, standard 0.035″ guidewire (Cordis) was used to remove the tunneled dialysis catheter and to insert therapeutic catheter Vertebral 5F (Cordis). Four metal coils Tornado (Cook Inc. Bloomington, IN) with a diameter of 6 and 7 mm were then implanted over 5F catheter into the mediastinal tissue canal under the protection of the balloon catheter. The last coil was placed in the ostial portion of the tissue canal. In the next step, a microcatheter Progreate 2.7 F (Terumo) was introduced to the tissue canal over the therapeutic 5F catheter. The embolization of the whole length of the tissue canal from the tip to the ostium filled with metal coils was performed with the use of Histoacryl glue (Braun) mixed with 30 % Lipidol Ultra Fluid (Guerbet) (Fig. 2). Then, the microcatheter was removed and the therapeutic catheter was pulled back. Simultaneously, balloon protection was removed. The follow-up venography showed no contrast extravasation into the mediastinum and normal opacification of the left innominate vein was demonstrated (Fig. 3). The patient remained hemodynamically stable through all the procedure and was dismissed home 1 week later.

Our goal was to combine the properties of synthetic fibers of Tornado embolization coils (thrombogenicity, promotion of clot formation within the mediastinal canal) with the great adhesiveness of Histoacryl glue to the synthetic fibers of Tornado platinum coils. We also utilized tornado-like configuration to maximize coil exposure to the cross-section of mediastinal canal lumen to disrupt blood flow. Therefore, our purpose was to deploy the proximal coil relatively close to the ostium of mediastinal canal, in close proximity of the wall of the damaged vessel to promote clotting and to minimize the risk of the migration of glue into the lumen of left innominate vein. Conformance to the tissue canal and radial force of the proximal coil at the site of ostium were supposed to anchor the glue and to minimize the risk of its migration into the vessel lumen.

The potential dislodgement of both proximal coil and Histoacryl glue into one of the small branches of pulmonary arteries would not most probably have resulted in clinically relevant consequences. Patients with peripheral pulmonary embolism relatively often demonstrate no clinical symptoms. Nevertheless, coil migration to the pulmonary arteries could be potentially fatal. To counteract such sequelae, we used a protective balloon during the procedure of the embolization of mediastinal canal, which was of particular relevance when deploying the proximal coil and injecting the glue into the adjacent portion of mediastinal canal. The protective balloon catheter also served to prevent bleeding from the perforation site following the removal of the dialysis catheter.

If the procedure of embolization was not successful, we would try to inject thrombin into the mediastinal canal with additional balloon catheter protection [6]. Finally, if this step failed, we would consider stent-graft implantation. However, the localization of the vessel damage and circulatory failure were the factors that would jeopardize the thrombosis of stent-graft lumen. In fact the proximal coil migrated minimally distally during the procedure contrary to our primary intention. However, we are still of the opinion that the presence of the proximal coil close to the site of perforation proved beneficial, because it minimized the proximal migration of glue. We were aware of the possibility of such migration; therefore, we secured the site of perforation using the protective balloon.
